# *Staphylococcus aureus* and *Escherichia coli* have disparate dependences on KsgA for growth and ribosome biogenesis

**DOI:** 10.1186/1471-2180-12-244

**Published:** 2012-10-24

**Authors:** Heather C O’Farrell, Jason P Rife

**Affiliations:** 1 Department of Physiology and Molecular Biophysics, Virginia Commonwealth University, Richmond, VA, USA; 2 Institute for Structural Biology and Drug Discovery, Virginia Commonwealth University, Richmond, VA, USA

**Keywords:** KsgA, Ribosome biogenesis, Staphylococcus aureus, Escherichia coli, Methyltransferase

## Abstract

**Background:**

The KsgA methyltransferase has been conserved throughout evolution, methylating two adenosines in the small subunit rRNA in all three domains of life as well as in eukaryotic organelles that contain ribosomes. Understanding of KsgA’s important role in ribosome biogenesis has been recently expanded in *Escherichia coli*; these studies help explain why KsgA is so highly conserved and also suggest KsgA’s potential as an antimicrobial drug target.

**Results:**

We have analyzed KsgA’s contribution to ribosome biogenesis and cell growth in *Staphylococcus aureus*. We found that deletion of *ksgA* in *S. aureus* led to a cold-sensitive growth phenotype, although KsgA was not as critical for ribosome biogenesis as it was shown to be in *E. coli*. Additionally, the *ksgA* knockout strain showed an increased sensitivity to aminoglycoside antibiotics. Overexpression of a catalytically inactive KsgA mutant was deleterious in the knockout strain but not the wild-type strain; this negative phenotype disappeared at low temperature.

**Conclusions:**

This work extends the study of KsgA, allowing comparison of this aspect of ribosome biogenesis between a Gram-negative and a Gram-positive organism. Our results in *S. aureus* are in contrast to results previously described in *E. coli,* where the catalytically inactive protein showed a negative phenotype in the presence or absence of endogenous KsgA.

## Background

Ribosome biogenesis in bacteria involves a small number of extra-ribosomal biogenesis factors [1]. Depletion or loss of many of these factors leads to impaired ribosome assembly, and in many cases leads to growth defects or even loss of virulence in pathogenic bacteria. Understanding ribosome biogenesis in bacteria is an active field of study; the bulk of this work has taken place in the model organism *Escherichia coli*, a Gram-negative γ-proteobacterium, while lesser study has occurred in other organisms, principally the Gram-positive organism *Bacillus subtilis*. One ribosome biogenesis factor in particular, KsgA, has been studied intensively for many years in *E. coli*. KsgA dimethylates each of two adenosines in the 3’-proximal helix (helix 44) of the small subunit rRNA [2] and serves as an important checkpoint in the assembly of the 30S subunit [3]. Cells lacking functional KsgA are often disadvantaged for growth when compared to wild-type cells. Specifically, knockout or mutation of *ksgA* in the organisms *E. coli*[3]*, B. subtilis*[4], *Mycobacterium tuberculosis*[5], *Yersinia pseudotuberculosis*[6], *Chlamydia trachomatis*[7] and *Erwinia amylovora*[8] is deleterious to cell growth, producing strains that either grow slower than or are unable to compete efficiently with wild-type strains. In addition, knockout of *ksgA* in *Y. pseudotuberculosis* confers an attenuated virulence phenotype on the knockout strain [6]; inactivating mutations of *ksgA* in the plant pathogen *E. amylovora* decrease virulence [8].

A key observation to come out of the body of work on KsgA is that overexpression of catalytically inactive KsgA produces a dominant negative phenotype, being deleterious to both ribosome biogenesis and cell growth, thus suggesting KsgA might serve as a potential antimicrobial drug target [3]. In this context KsgA and its role in ribosome biogenesis and growth have been studied most extensively in *E. coli*. While *ksgA* gene knockouts have been tangentially studied in other organisms, no systematic study has been made of KsgA and its role in ribosome biogenesis and growth in another bacterial organism. In order to expand our knowledge of this system, we have extended studies of KsgA into the important Gram-positive human pathogen *Staphylococcus aureus*.

## Results

### Knockout of *ksgA* leads to a cold-sensitive phenotype

**Figure 1:**
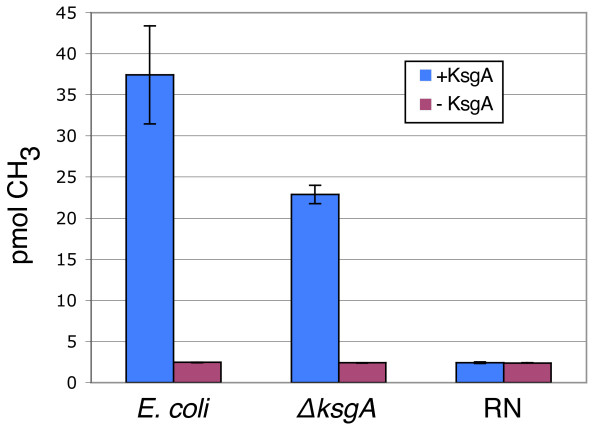
**Activity assay.** Experiments were performed in triplicate; error bars indicate standard deviation.

To investigate the role KsgA plays in ribosome assembly and growth we generated an in-frame deletion of the *ksgA* gene in the *S. aureus* strain RN4220. The knockout strain was resistant to the antibiotic kasugamycin (Table 1); this resistant phenotype is also seen in *E. coli*. We confirmed the loss of KsgA activity in the cell by assaying purified 30S ribosomal subunits from both the wild-type (RN) and the knock-out (Δ*ksgA*) strains for their ability to be methylated by exogenously added KsgA (Figure 1). As expected, subunits from the RN strain could not be further methylated by recombinant *E. coli* KsgA, while subunits from the Δ*ksgA* strain could be efficiently methylated, albeit not to the same extent as *E. coli* 30S subunits. In addition to confirming the gene deletion, this experiment demonstrated that the structural requirements for KsgA binding to and methylating the small ribosomal subunit are conserved between *E. coli* and *S. aureus*.

**Table 1:** **Antibiotic resistance of RN4220 and Δ*ksgA*strains**

	**MIC (μg/ml)**	
	**RN4220**	Δ***ksgA***
Kasugamycin	800	>3200
Kanamycin	4	2
Paromomycin	4	2
Streptomycin	16	16

Next we compared the growth rates of the Δ*ksgA* strain and the parental RN cells. We grew both strains in liquid media at a variety of temperatures (Additional file 1) and calculated the doubling times for each strain, shown in Table 2. The strains grew at similar rates at 30°C, 37°C, and 42°C. However, at the lower temperatures of 25°C and 15°C the Δ*ksgA* strain grew significantly slower than the RN strain. We can conclude from these data that while knockout of *ksgA* does not affect cell growth using our test conditions at and around human physiological temperatures the cells become cold-sensitive upon loss of KsgA.

**Table 2:** Doubling times of RN4220 and ΔksgA strains

	**Doubling time (min)**	
	**RN4220**	**Δ*ksgA***
15°C	408.2 ± 18.2	473.0 ± 17.2
25°C	82.1 ± 4.1	93.4 ± 2.0
30°C	48.5 ± 0.6	50.2 ± 2.2
37°C	39.2 ± 1.8	39.4 ± 1.7
45°C	50.6 ± 1.5	54.3 ± 3.5

**Figure 2:**
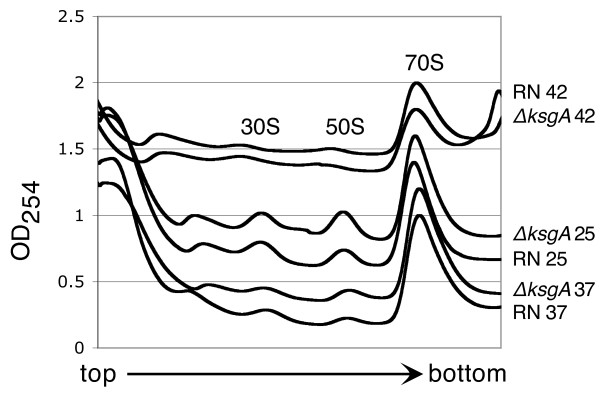
**Polysome analysis of the RN4220 and ΔksgA strains.** Each chromatogram was normalized to a value of 1.0 for the 70S peak; successive chromatograms were offset by 0.2 on the y-axis.

We then performed polysome analysis of the ribosomal particles of both strains to ascertain the effects of *ksgA* knockout on ribosome biogenesis. In these experiments ribosomal material is separated into mature, functional 70S ribosomes and free 30S and 50S subunits. In this way we can visualize increases in immature subunits as a portion of the total ribosomal material. As shown in Figure 2, knockout of *ksgA* did not result in a significant increase in relative amounts of free 30S subunits. Polysome profiles of the RN and Δ*ksgA* strains were similar at 42°C, 37°C, and 25°C; the proportion of free subunits increased with lowering temperature in both strains.

Our laboratory previously observed that knockout of *ksgA* in *E. coli* led to a difference in sensitivity to aminoglycoside antibiotics [9]. Specifically, the Δ*ksgA* strain was more sensitive to the 4,6 class of aminoglycosides and less sensitive to 4,5-aminoglycosides, with no change in sensitivity to the aminoglycoside streptomycin. We performed a similar experiment in *S. aureus*, growing the RN and Δ*ksgA* strains on increasing amounts of the antibiotics kanamycin (a 4,6 aminoglycoside), paromomycin (a 4,5-aminoglycoside) and streptomycin (Table 1). The Δ*ksgA* strain was more sensitive to both kanamycin and paromomycin, with no change in sensitivity to streptomycin.

### Overexpression of catalytically inactive KsgA is deleterious

Overexpression of KsgA, as well as a catalytically inactive mutant of KsgA, was deleterious to *E. coli* growth rates under a variety of conditions [3]. In order to see if these results extended to *S. aureus* we cloned the *ksgA* gene from the RN4220 strain and constructed the equivalent mutation, E79A. We expressed both WT and E79A protein in RN and Δ*ksgA* cells, using the empty vector (pCN) as a control. Growth experiments were performed as in the previous section (Additional file 2), except that cells were grown in the presence of erythromycin (for plasmid maintenance) and CdCl_2_ (for protein induction). Under these conditions, the difference in growth rate between the RN and Δ*ksgA* cells expressing the empty vector was not significant, even at 25°C. Doubling times for each strain are shown in Table 3.

**Table 3:** **Doubling times of RN4220 and Δ*ksgA*strains containing pCN constructs**

	**Doubling time (min)**	
	**25°C**	**37°C**
RN4220 pCN51	95.5 ± 13.8	40.5 ± 2.7
pCN-WT	94.9 ± 11.0	39.6 ± 2.4
pCN-E79A	92.6 ± 9.5	39.2 ± 4.7
ΔksgA pCN51	106.1 ± 11.6	41.4 ± 2.7
pCN-WT	100.0 ± 8.0	38.3 ± 2.5
pCN-E79A	111.3 ± 11.5	51.0 ± 2.3

Overexpression of wild-type KsgA did not affect cell growth under any of the conditions we tested. Overexpression of the E79A mutant in cells lacking *ksgA* had a negative impact on doubling time, but only in the absence of WT enzyme. This effect was seen at 37°C but not at 25°C. In the RN strain, which expresses endogenous KsgA, overexpression of mutant protein did not significantly affect cell growth.

**Figure 3:**
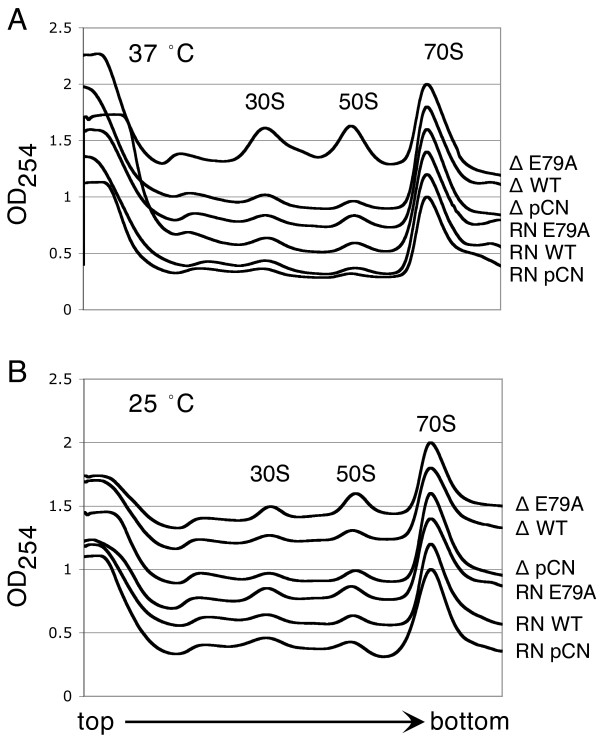
**Polysome analysis of the pCN51 strains.** Each chromatogram was normalized to a value of 1.0 for the 70S peak; successive chromatograms were offset by 0.2 on the y-axis. **A**) Cells grown at 37°C. **B**) Cells grown at 25°C.

We next asked if there were any abnormalities in ribosome biogenesis in cells overexpressing WT or mutant KsgA protein. In *E. coli* overexpression of WT protein led to accumulation of immature 30S subunits even when there was no measurable effect on cell growth, and overexpression of the inactive mutant, E66A, resulted in significant effects on ribosome biogenesis in all cases. In *S. aureus*, overexpression of either WT or E79A protein had very little effect on ribosome biogenesis under any conditions tested (Figure 3), with one exception. The *S. aureus* Δ*ksgA* strain overexpressing the E79A mutant protein showed an increase in free subunits relative to the total ribosomal material when grown at 37°C but not at 25°C.

## Discussion

The existence of the *ksgA* gene was established about forty years ago in *E. coli*[10]. It was shown to be the sole methyltransferase that converts two adjacent 16S rRNA adenosines (A1518 and A1519, *E. coli* numbering) into N^6^,N^6^-dimethyladenosines [2], modifications that appeared to hold wide phylogenetic distribution. It is now known that those modifications and the responsible methyltransferase are all but universally conserved throughout life, thus making KsgA (known as Dim1 in eukaryotes and archaea) a genetic element of the last universal common ancestor. This level of conservation, coupled with the knowledge that KsgA can be dispensed with in several bacteria, albeit with obvious growth defects [3-8], formed the basis of a sharp paradox. If KsgA was not essential, why was it universally conserved? Since evolution is not sentimental, the cellular importance of KsgA and Dim1 was certain but remained to be discovered. In time the stated paradox has partially unraveled. In *Saccharomyces cerevisiae* (and most likely other eukaryotic organisms) Dim1 is an important member of the rRNA processome, and loss of Dim1 leads to the accumulation of aberrant rRNA species at the expense of functional ribosomes [11]. In *E. coli*, KsgA serves as a gate-keeper to prevent improperly assembled pre-30S subunits from entering the translation cycle [3]. Under normal conditions, KsgA only provides modest benefit to 30S maturation and function. However, KsgA’s importance becomes clear under stress conditions, such as growth at cold temperature.

In this work, we sought to define the importance of KsgA to the survivability of the human pathogen *S. aureus* and to compare our results to those in the model organism *E. coli*. Somewhat surprisingly, we found that *S. aureus* has a lesser reliance on KsgA under the conditions tested. In *E. coli*, overexpression of KsgA rescued the cold-sensitive phenotype of Δ*ksgA* cells at low temperature but was deleterious for cell growth at 37°C in both knockout and parental cells. Overexpression of a catalytically inactive mutant of KsgA, E66A, was deleterious in both strains at both temperatures, even in the presence of endogenous WT protein [3]. We showed that in *S. aureus* the *ksgA* knock-out strain displayed a slow growth phenotype at low temperature when compared to the parental strain, similar to results in *E. coli*. However, unlike in *E. coli*, catalytic inactivation of KsgA’s enzymatic function has only mild phenotypic effects, and these effects are not dominant in the presence of WT KsgA. It is noteworthy that the negative growth effect was seen at 37°C but not at 25°C. This result was unexpected, both because *ksgA* knockout led to cold sensitivity and because negative effects in *E. coli* were exacerbated at low temperature; however, it is possible that growth at the lower temperature results in lower expression of the mutant protein and therefore a smaller negative effect.

In *S. aureus*, KsgA also appears to be less critical for the assembly of mature ribosomes. Experiments in *E. coli* showed that loss or inactivation of KsgA had obvious effects on ribosome biogenesis even under conditions where a growth phenotype was not apparent [3]. In other words, ribosome biogenesis is sensitive to disruptions in KsgA function that don’t affect overall cell growth. We did not see this effect in *S. aureus*; knockout or inactivation of KsgA resulted in, at most, slight disruption of polysome profiles even under conditions where cell growth was slowed.

On the basis of the data presented here, it would appear that in *S. aureus* KsgA holds less promise as a drug target than in *E. coli*. However, we did observe that knockout of *ksgA* rendered *S. aureus* marginally more sensitive to clinically used aminoglycoside antibiotics, similar to results seen in *E. coli*. A1518 and A1519 are located distal to the aminoglycoside binding site on the small ribosomal subunit; we therefore hypothesize that effects on antibiotic sensitivity are indirect, likely caused either by conformational or dynamic changes that are propagated from the site of KsgA methylation to the aminoglycoside binding site. This experiment highlights an additional difference between *E. coli* and *S. aureus* ribosomes. While lack of methylation by KsgA leads to increased sensitivity to the 4,6 class of aminoglycosides in both organisms, we see opposite effects on 4,5 aminoglycoside sensitivity. Both the KsgA target site and the aminoglycoside binding site are among the most highly conserved rRNA sequences; it is thus intriguing that distinct effects are seen between the two organisms.

Although ribosome biogenesis has not been well-studied outside of the model organisms *E. coli* and, to a much lesser extent, *B. subtilis*, it is possible that reported differences in ribosome biogenesis between Gram-negative and Gram-positive organisms are representative of an evolutionary divergence between the two groups of bacteria. One such difference is the case of the ribonuclease RNase III. RNase III is an endonuclease that is involved in processing of the pre-rRNA transcript in both *E. coli* and *B. subtilis*. However, this enzyme is strictly essential in *B. subtilis* but not in *E. coli*[12]. Additionally, inactivation of RNase III has different effects on the maturation of 16S rRNA in the two organisms [12]. Further work is required to demonstrate whether these results are more broadly applicable in other bacterial species. Our work suggests differences in ribosome biogenesis between *E. coli* and *S. aureus*; it remains to be seen if the differing reliance on KsgA can be defined by a phylogenetic Gram-positive/Gram-negative split.

KsgA plays a key role in ribosome biogenesis in *E. coli*, which cannot be separated from its methyltransferase function [3]. Further evidence of KsgA’s significance in Gram-negative organisms comes from virulence studies in pathogenic organisms. Disruption of *ksgA* in *Y. pseudotuberculosis* confers an attenuated virulence phenotype on the knockout strain [6], and this attenuated strain confers protection against subsequent challenge with the wild-type strain [13]. Additionally, mutation of *ksgA* in the plant pathogen *E. amylovora* decreases virulence [8] and disruption of KsgA in *S.* Enteriditis reduces invasiveness [14]. These studies affirm that KsgA may be a novel drug target in Gram-negative organisms.

Studies on KsgA’s role in virulence have not been done in Gram-positive organisms, although in addition to the modest growth defects seen in the *S. aureus* Δ*ksgA* strain disruption of the *ksgA* gene in the Gram-negative *Mycobacterium tuberculosis* was shown to negatively affect bacterial growth on solid media [5]. It should be noted that disruption of *ksgA* in *Y. pseudotuberculosis* produced only a slight growth defect and allowed the bacteria to survive in infected mice, even though the strain was not as virulent as the wild-type strain [6]. Likewise, *E. amylovora* mutants showed reduced virulence despite only small growth defects *in vitro* and the ability to grow in infected tissue [8]. Further studies will be required to show whether KsgA is similarly correlated with virulence in Gram-positive organisms.

## Conclusions

Given the vital role that the ribosome plays in the cell, it is unsurprising that it is an important target for antibiotic drugs [15]. Although current antibiotic strategies are directed at the functioning of the ribosome, it has been suggested that the ribosome assembly presents a target for novel drug discovery [16]. In support of this hypothesis, knockout of the non-essential ribosome biogenesis factors KsgA and YjeQ, a small-subunit associated GTPase, has been shown to affect bacterial virulence [6,8,17]. Therefore, a full understanding of these and other ribosome biogenesis factors in a variety of organisms is critical.

We have extended the study of KsgA into *S. aureus* and found that KsgA is not as critical for bacterial growth and ribosome biogenesis as was previously shown to be the case in *E. coli*, although the Δ*ksgA* knockout does have some negative effects. Additionally, overexpression of the catalytically inactive mutant did not have a dominant effect on growth or ribosome biogenesis in the presence of wild-type protein. Although knockout and mutation of KsgA did not lead to severe growth defects, work in *Y. pseudotuberculosis* and *E. amylovora* suggests that small growth defects *in vitro* may correlate with larger effects on virulence. Many researchers have suggested that targeting virulence may be a better strategy for antimicrobial therapy than targeting cell growth or viability [18,19]. We believe that further research on the role of KsgA in the virulence of *S. aureus* and other pathogens will prove instructive and may provide a viable drug development target.

## Methods

### Strains and plasmids

The RN4220 strain, the pCN51 expression vector, and genomic DNA from *S. aureus* strain 8325 were gifts from Dr. Gordon Archer, Virginia Commonwealth University. The pMAD shuttle vector for knockout of *ksgA* was a gift from Dr. Gail Christie, Virginia Commonwealth University.

We constructed a *ksgA* knockout of the *S. aureus* RN4220 strain according to the method of Arnaud *et al*[20]. Allelic replacement was performed using the primers in Additional file 3; chromosomal knockout was confirmed by PCR.

The *ksgA* gene was amplified from genomic DNA from *S. aureus* strain 8325, adding a ribosome binding sequence to ensure translation; primers used for cloning are shown in Additional file 3. The resulting fragment was subcloned into the pCN51 expression vector to produce pCN-WT. Mutagenesis was performed on this plasmid according to the Stratagene Quikchange protocol to produce pCN-E79A. The pCN51 constructs were transformed into strain RN4220 (RN) and the *ksgA* knockout strain (Δ*ksgA*) by electroporation. Expression of active protein from the pCN51-KsgA plasmid was confirmed in the Δ*ksgA* strain by the kasugamycin resistance assay (Additional file 4), as well as by showing that 30S subunits purified from this strain were not able to be further methylated by KsgA (Additional file 5).

### Antibiotic resistance assay

Cells were grown in tryptic soy broth (TSB) at 37°C overnight; saturated culture was subcultured to an OD_600_ of 0.02 in TSB and grown with shaking at 225 rpm to an OD_600_ of 0.6-0.8. The culture was then diluted 1:100 and plated onto varying concentrations of antibiotic. Plates were grown at 37°C overnight; the minimal inhibitory concentration (MIC) was read as the lowest concentration of antibiotic which prevented growth.

### Activity assay

30S subunits were prepared from the *S. aureus* RN4220 and Δ*ksgA* strains as well as from an *E. coli* wild-type strain. Cells were grown in TSB (*S. aureus*) or LB (*E. coli*) to mid-log phase. Cells were harvested and the cell pellet resuspended in Buffer I (50 mM Tris, pH 7.4, 100 mM NH_4_Cl, 10 mM MgOAc, and 6 mM β-mercaptoethanol). Glass beads (0.090-0.135 mm, Thomas Scientific) were added to a final concentration of 1 mg/μl and the suspension was vortexed for 10 minutes. The lysates were cleared by centrifugation at 4°C, layered onto 1.1 M sucrose in Buffer II (50 mM Tris, pH 7.4, 1 M NH_4_Cl, 10 mM MgOAc, and 6 mM β-mercaptoethanol), and spun in a 70Ti rotor at 35,000 rpm for 22 hours at 4°C. The pellet of ribosomal material was resuspended in Buffer III (50 mM Tris, pH 7.4, 500 mM NH_4_Cl, 2 mM MgOAc, and 6 mM β-mercaptoethanol) and loaded onto a 10-40% sucrose gradient in Buffer III. The gradients were spun in an SW-28 rotor at 19,000 rpm for 17 hours at 4°C and 30S fractions were collected, dialyzed into Buffer K (50 mM Tris, pH 7.4, 500 mM NH_4_Cl, 2 mM MgOAc, and 6 mM β-mercaptoethanol) and stored at -80°C. *E. coli* KsgA was purified as previously described; activity assays were performed as previously described [21].

### Growth experiments

Cells were grown in TSB at 37°C overnight; cultures of strains transformed with pCN constructs included erythromycin (10 μg/ml). Saturated culture was subcultured to an OD_600_ of 0.1 in TSB; media contained cadmium (2 μM) and erythromycin (10 μg/ml) for experiments with the pCN constructs. Cells were incubated with shaking (225 rpm) and the OD_600_ was monitored. Data were fit to an exponential growth model using the Graphpad Prism software and doubling times were calculated from the equation Y = Y_0._ × e^K× X^.

### Polysome analysis

Cells were grown in TSB, containing cadmium (2 μM) and erythromycin (50 μg/ml) as appropriate, to mid-log phase. Cells were harvested and the cell pellet resuspended in Buffer PA μg/ml (20 mM Tris, pH 7.8, 100 mM NH_4_Cl, 10 mM MgCl_2_, and 6 mM β-mercaptoethanol). Glass beads (0.090-0.135 mm, Thomas Scientific) were added to a final concentration of 1 mg/μl and the suspension was vortexed for 10 minutes. The lysates were cleared by centrifugation at 4°C and loaded onto a 10-40% sucrose gradient in Buffer PA. The gradients were spun in an SW-28 rotor at 19,000 rpm for 17 hours at 4°C. Gradients were analyzed at 254 nm using a Biocomp Piston Gradient Fractionator with a BIORAD Econo UV Monitor with a Full Scale of 1.0. Data were recorded using DataQ DI-158-UP data acquisition software and the 70S peaks were then normalized to 1.

## Competing interests

The authors declare that they have no competing interests.

## Authors’ contributions

HCO carried out all experiments and drafted the manuscript. JPR conceived of the study, participated in its design and coordination, participated in construction of the knockout strain, and helped to draft the manuscript. All authors read and approved the final manuscript.

## Supplementary Material

Additional file 1 **Growth curves of RN and Δ*ksgA *strains.** Data represent experiments performed in triplicate; error bars indicate standard deviation. Click here for file

Additional file 2 **Growth curves of pCN constructs.** Data represent experiments performed in triplicate; error bars indicate standard deviation. Click here for file

Additional file 3 Primers used in knockout construction, KsgA cloning, and mutagenesis. Click here for file

Additional file 4 Antibiotic resistance of RN4220, ΔksgA, and ΔksgA + pCN51-KsgA strains. Click here for file

Additional file 5 **Activity assay.** Experiments were performed in triplicate; error bars indicate standard deviation. Click here for file
