# Supplementary figures and images for: Staphylococcus aureus and Escherichia coli have disparate dependences on KsgA for growth and ribosome biogenesis
Source: BMC Microbiol. 2012 Oct 24;12:244. doi: 10.1186/1471-2180-12-244 (PMC3534330; doi:10.1186/1471-2180-12-244)

15 °C

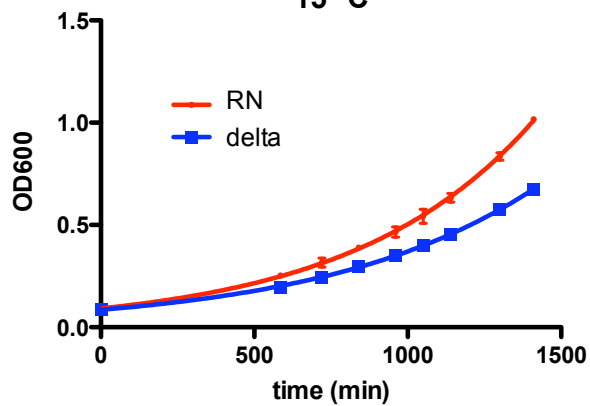

25 °C

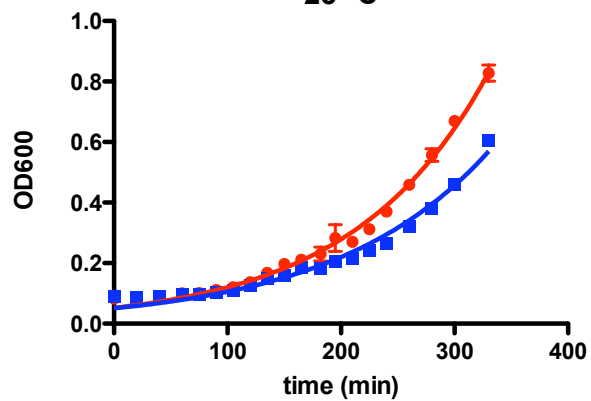

30 °C

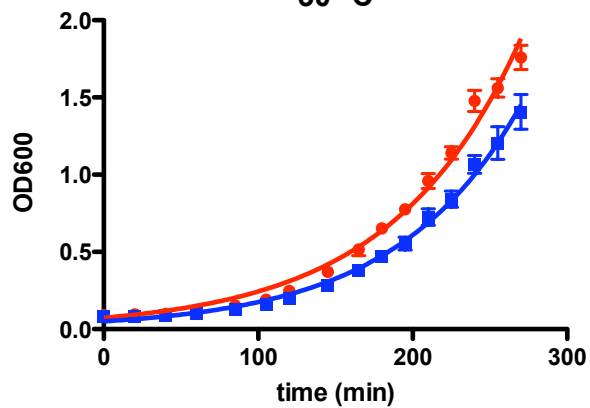

37 °C

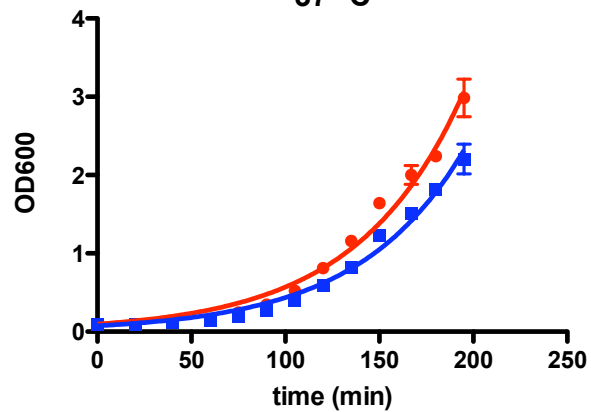

45 °C

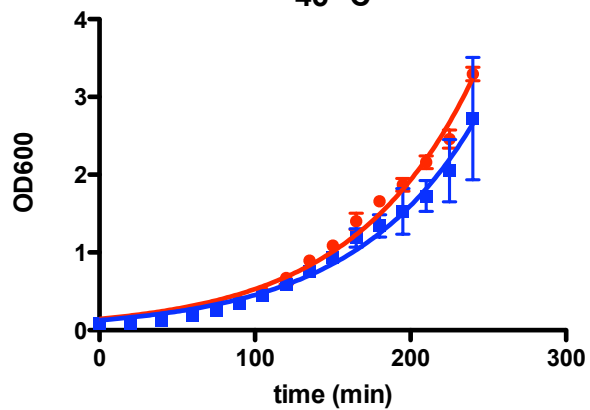

Supplement: Additional file 1 — Growth curves of RN and ΔksgA strains. Data represent experiments performed in triplicate; error bars indicate standard deviation. [file 1471-2180-12-244-S1.pdf]

25 °C

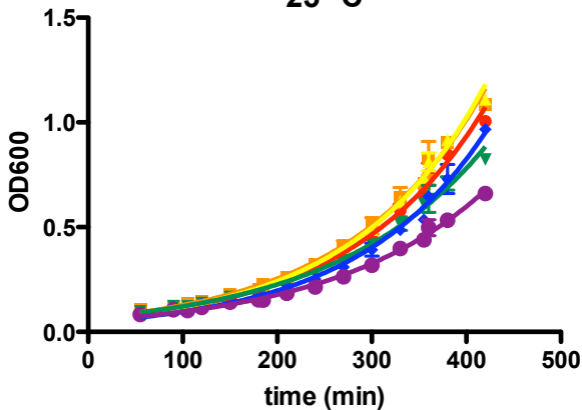

37 °C

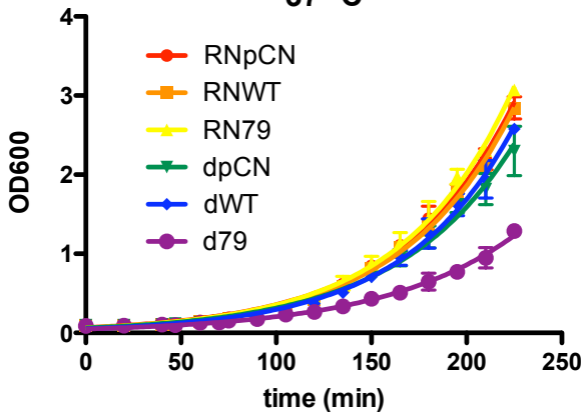

Supplement: Additional file 2 — Growth curves of pCN constructs. Data represent experiments performed in triplicate; error bars indicate standard deviation. [file 1471-2180-12-244-S2.pdf]

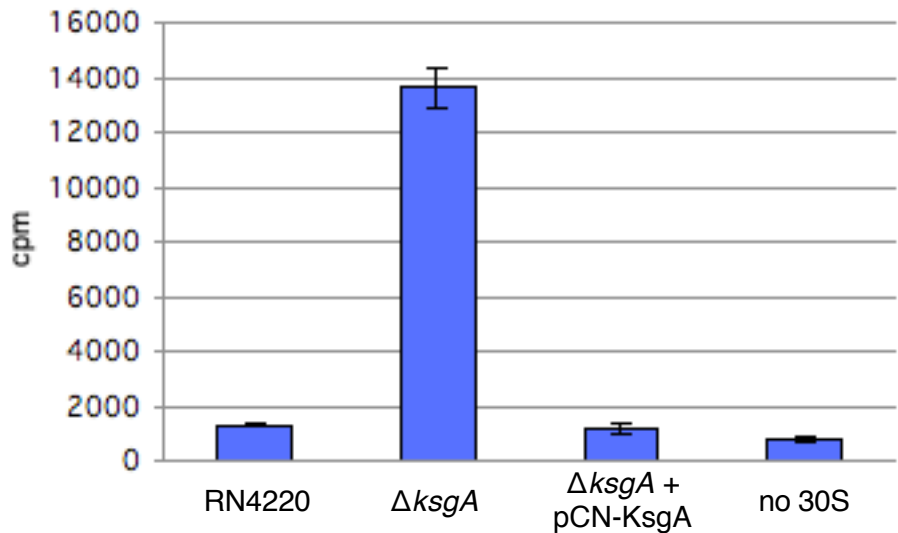

Supplement: Additional file 5 — Activity assay. Experiments were performed in triplicate; error bars indicate standard deviation. [file 1471-2180-12-244-S5.pdf]
