# Supplementary material for: Staphylococcus aureus and Escherichia coli have disparate dependences on KsgA for growth and ribosome biogenesis
Source: BMC Microbiol. 2012 Oct 24;12:244. doi: 10.1186/1471-2180-12-244 (PMC3534330; doi:10.1186/1471-2180-12-244)
Supplement: Additional file 3 — Primers used in knockout construction, KsgA cloning, and mutagenesis. [file 1471-2180-12-244-S3.pdf]

Primer name	Sequence
UpForNcoI	5’- CGTCCATGGGCATAGCTTTAGTGGTTCTCCAG -3’
UpRevXhoI	5’- GCGGCTCGAGCATTTTCACTTCCTCATTCATCTTCTAAAG-3’
DownForXhoI	5’-GCGGCTCGAGGACAAAGCAAAGCACTATTGTTAAAATTTAAATTTTG-3’
DownRevNcoI	5’-GCACCATGGCTTAACGTTGCTGCTGCATC-3’
SaKsgARBSFor	5’- GAACCGTCGACAATGAGGAAGTGAAAATGTTGG-3’
SaKsgARev	5’-GCTTCCGGATCCCTTTGTCAATCATTTAATTTTCTAATTGAG-3’
SaKsgAE79AFor	5'- GCTAAAAGAGTATTGGCATTTGCAATTGATCAACGTTTAATACC -3'
SaKsgAE79ARev	5'- GGTATTAAACGTTGATCAATTGCAAATGCCAATACTCTTTTAGC -3'
