# Supplementary material for: Staphylococcus aureus and Escherichia coli have disparate dependences on KsgA for growth and ribosome biogenesis
Source: BMC Microbiol. 2012 Oct 24;12:244. doi: 10.1186/1471-2180-12-244 (PMC3534330; doi:10.1186/1471-2180-12-244)
Supplement: Additional file 4 — Antibiotic resistance of RN4220, ΔksgA, and ΔksgA + pCN51-KsgA strains. [file 1471-2180-12-244-S4.pdf]

	MIC (μg/ml ksg)
RN4220	800
Δ*ksgA*	>1600
Δ*ksgA* + pCN-KsgA	800
